# Genetic Determinants of Increased Body Mass Index Partially Mediate the Effect of Elevated Birth Weight on the Increased Risk of Atrial Fibrillation

**DOI:** 10.3389/fcvm.2021.701549

**Published:** 2021-08-06

**Authors:** Songzan Chen, Tian Xu, Fangkun Yang, Yao Wang, Kaijie Zhang, Guosheng Fu, Wenbin Zhang

**Affiliations:** ^1^Department of Cardiology, School of Medicine, Sir Run Run Shaw Hospital, Zhejiang University, Hangzhou, China; ^2^Key Laboratory of Cardiovascular Intervention and Regenerative Medicine of Zhejiang Province, Hangzhou, China; ^3^Department of Cardiology, School of Medicine, Second Affiliated Hospital, Zhejiang University, Hangzhou, China

**Keywords:** atrial fibrillation, birth weight, body mass index, causal association, genome-wide association study, mendelian randomization

## Abstract

**Background:** Although several observational studies have shown an association between birth weight (BW) and atrial fibrillation (AF), controversy remains. In this study, we aimed to explore the role of elevated BW on the etiology of AF.

**Methods:** A two-sample Mendelian randomization (MR) study was designed to infer the causality. The genetic data on the associations of single-nucleotide polymorphisms (SNPs) with BW and AF were separately obtained from two large-scale genome-wide association studies with up to 321,223 and 1,030,836 individuals, respectively. SNPs were identified at a genome-wide significant level (*p* <5 × 10^−8^). The inverse variance-weighted (IVW) method was employed to obtain causal estimates as our primary analysis. Sensitivity analyses with various statistical methods were applied to evaluate the robustness of the results, and multivariable MR analysis was conducted to determine whether this association was mediated by the body mass index (BMI).

**Results:** In total, 144 SNPs were identified as the genetic instrumental variables. MR analysis revealed a causal effect of elevated BW on AF (OR = 1.27, 95% CI = 1.14–1.40, *p* = 5.70 × 10^−6^). All the results in sensitivity analyses were consistent with the primary result. The effect of BW on AF was attenuated when adjusted for BMI (OR = 1.16, 95% CI = 1.01–1.33, *p* = 0.04).

**Conclusions:** This study indicated that elevated BW was significantly associated with increased lifelong risk of AF, which may be partially mediated by BMI.

## Introduction

Atrial fibrillation (AF) represents the most common sustained cardiac arrhythmia with significant morbidity and mortality, responsible for a substantial health care burden all over the world (1–3). The global number of individuals with AF was estimated to be 33.5 million in 2010, and accumulated evidence have suggested an increasing prevalence and incidence of AF during the recent years (3–5). Considerable effort has been made to manage this disease, whereas the benefit of eliminating the established AF remains limited (1, 2, 6). Further exploration of the AF pathophysiology and discovery of the new risk factors are warranted, since the efficient prevention is of great importance (7, 8).

Birth weight (BW) represents a well-established risk factor for ischemic heart disease (9–11). In addition, numerous studies have suggested that BW is also associated with several cardiovascular risk factors such as type 2 diabetes mellitus and hypertension (12, 13). However, it remains inconclusive whether BW is associated with the risk of AF. To our knowledge, only four previous studies have explored the association between BW and AF, with discordant results (14–17). Further investigation is warranted to reveal whether this association is causal (18).

Mendelian randomization (MR) analysis has been increasingly used to infer the causation (19, 20). MR exploits genetic variants, usually single-nucleotide polymorphisms (SNPs), as proxies for the exposure of interest (19, 20). Based on Mendel's second law, these SNPs were randomly allocated at conception, which could be thought as a natural randomized controlled trial (21). MR analysis is less susceptible to potential unmeasured confounders and reverse causation (19, 20). Two-sample MR is an extension of this methodology, which derives the genetic association data from two separate genome-wide association studies (GWASs) (22, 23). With the enlargement of the sample size, the statistical power is largely improved. In this study, we aim to systematically appraise the evidence of the causal association between BW and AF using the two-sample MR analysis.

## Methods

### Study Design

**Figure 1:**
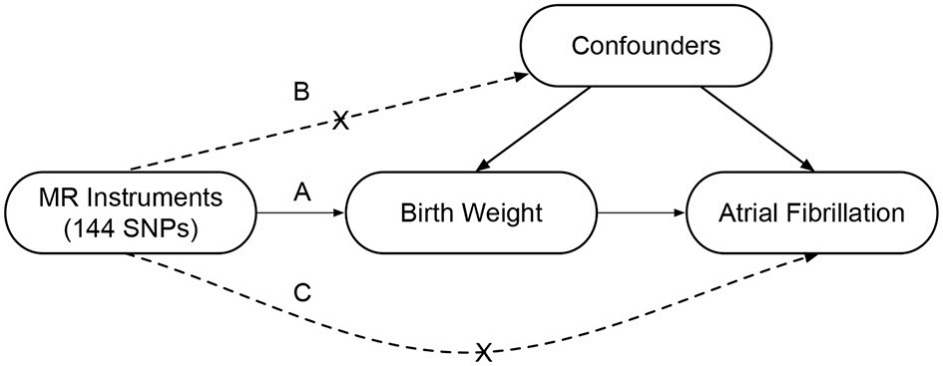
Conceptual framework of the two-sample Mendelian randomization study design. This method is based on three key assumptions as follows: **(A)** The SNPs should be associated with the birth weight; **(B)** The SNPs should be independent of the confounders; **(C)** The SNPs should be associated with the atrial fibrillation only via the birth weight. MR, Mendelian randomization; SNP, single-nucleotide polymorphism.

A two-sample MR study was designed to investigate the causal association between BW and the risk of AF (Figure 1). This method was based on three key assumptions (19). First, the genetic instrumental variables, i.e., SNPs, should be strongly associated with the BW. Second, the instrumental variables should be independent of confounders that may affect the association between the BW and the risk of AF. Third, the instrumental variables should be only associated with the risk of AF via BW.

### Data Sources

#### Exposure: BW

The exposure in this study was genetically predicted BW, in standard deviations (SDs). The SNPs that proxied for BW were extracted from the hitherto largest GWAS meta-analysis on BW using data from the Early Growth Genetics (EGG) Consortium and the UK biobank (*n* = 321,223) (24). This trans-ethnic (92.8% were European ancestry) meta-analysis consisted of three components: (i) 80,745 individuals of European ancestry from 35 studies within the EGG consortium, (ii) 12,948 individuals of diverse (non-European) ancestries from 9 studies within the EGG consortium, and (iii) 227,530 individuals of all ancestries from the UK Biobank (Supplementary Table 1). Premature and multiple births were excluded in most of the included studies.

#### Outcome: AF

The outcome in this study was AF. Summary statistics data on associations of SNPs with AF were derived from a recently published GWAS (*n* = 1,030,836) (25). This GWAS was the largest one on AF to date, which analyzed a total of 34,740,186 genotyped SNPs on up to 60,620 cases and 970,216 controls from six resources (Supplementary Table 2). The majority (98.6%) of the individuals were of European ancestry. AF was mainly diagnosed according to the International Classification of Diseases (ICD-9 and ICD-10).

### Statistical Analysis

#### Selection and Validation of Instrumental Variables (SNPs)

To ensure a close relationship between the genetic instrumental variables and BW, SNPs were identified at a genome-wide significant level (*p* <5 × 10^−8^) from the corresponding GWAS summary dataset. To check for correlations between each SNP, the pairwise-linkage disequilibrium (LD) was calculated using LD-Link based on European (https://ldlink.nci.nih.gov/) (26). When *r*^2^ > 0.001, only the SNP with lower *p*-value was retained. In addition, the effects of SNPs on AF were obtained from the corresponding dataset. If the specified SNP was not available for AF, a highly correlated SNP (*r*^2^ > 0.8) was selected for proxy (27). Additionally, any palindromic SNPs were removed from our analysis. Finally, F statistic was calculated for each SNP in order to detect whether this SNP was valid (*F* > 10) or not.

#### Primary MR Analysis

The inverse variance-weighted (IVW) method was employed to evaluate the causal association between BW and AF in this study as our primary analysis (28). Specifically, the causal effect of each SNP was estimated using the Wald estimator, and the relevant standard error was calculated using the Delta method (28). Then the IVW method was performed to meta-analyze each Wald ratio. Results were presented as odds ratios (ORs) with 95% confidence intervals (CIs) of AF per SD increased BW. The association of each SNP with BW was further plotted against its effect on AF. In addition, a power calculation was carried out using an online web-based tool named mRnd (https://shiny.cnsgenomics.com/mRnd/) (29). In this study, the statistical power was required to be at least 80%.

#### Sensitivity Analysis and Pleiotropy Assessment

In the follow-up sensitivity analyses, the maximum likelihood, simple median, weighted median (30), simple mode, weighted mode, MR-Egger (31), and Mendelian Randomization Pleiotropy Residual Sum and Outlier (MR-PRESSO) (32) methods were applied to test the robustness of our primary analysis. These methods were more robust for SNPs with potential pleiotropy. Subsequently, a leave-one-out analysis was conducted to determine whether the estimated causal effect was disproportionately affected by a single SNP. MR-Egger intercept test was performed to evaluate the potential directional pleiotropy. When the *p*-value of intercept was larger than 0.05, no horizontal pleiotropy existed. Additionally, a funnel plot was generated to visually inspect the pleiotropy, in which symmetry provided evidence against directional pleiotropy (33).

#### Multivariable MR Analysis

To determine whether the association between BW and AF was mediated or confounded by anthropometric traits, we investigated the effect of BW on AF conditional on body mass index (BMI) using multivariable MR analysis. The summary statistics data on associations of SNPs with BMI were obtained from a large GWAS and included about 700,000 individuals of European ancestry (34).

All the analyses were implemented using the “MendelianRandomization” and “TwoSampleMR” R packages in R (version 3.6.2) software environment (35, 36).

### Ethics Approval

Our study only made use of publicly available data, and hence, no additional ethics approval was required.

## Results

In total, 229 SNPs were obtained from the GWAS dataset of BW, which achieved the genome-wide significance (*p* <5 × 10^−8^). After exclusion of the correlated SNPs, 146 SNPs were retained. Among these SNPs, rs12623454, and rs6040076 were palindromic, which may cause ambiguity in the strand direction. Therefore, 144 SNPs were selected as genetic instrumental variables in our analyses. All the selected SNPs were valid (*F* > 10), and their characteristics and associations with BW and AF are shown in Supplementary Table 3.

### The Association Between BW and AF

**Figure 2:**
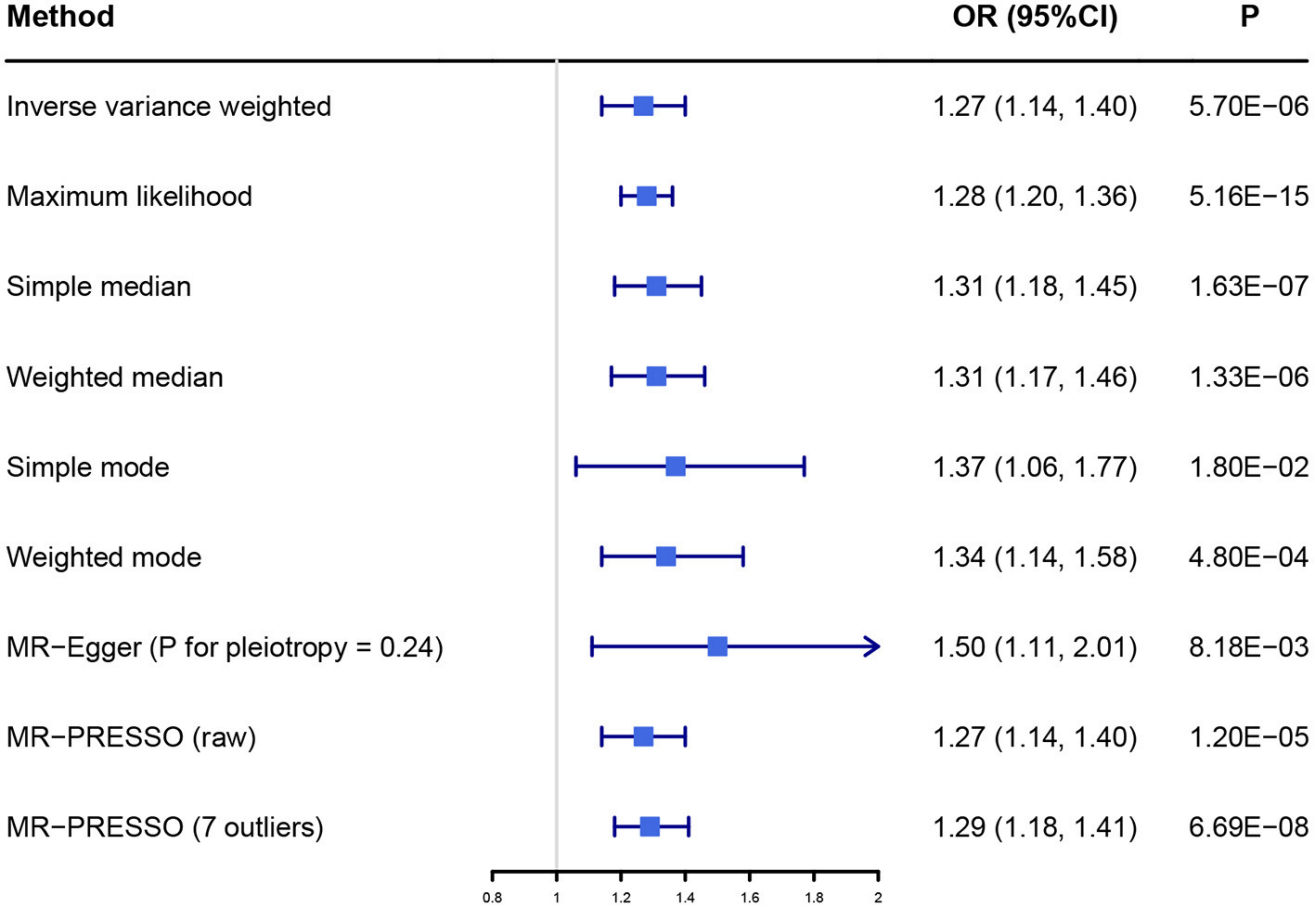
The association between birth weight and atrial fibrillation investigated with different statistical methods. OR, odds ratio; CI, confidence interval; MR, Mendelian randomization; MR-PRESSO, Mendelian Randomization Pleiotropy Residual Sum and Outlier.

**Figure 3:**
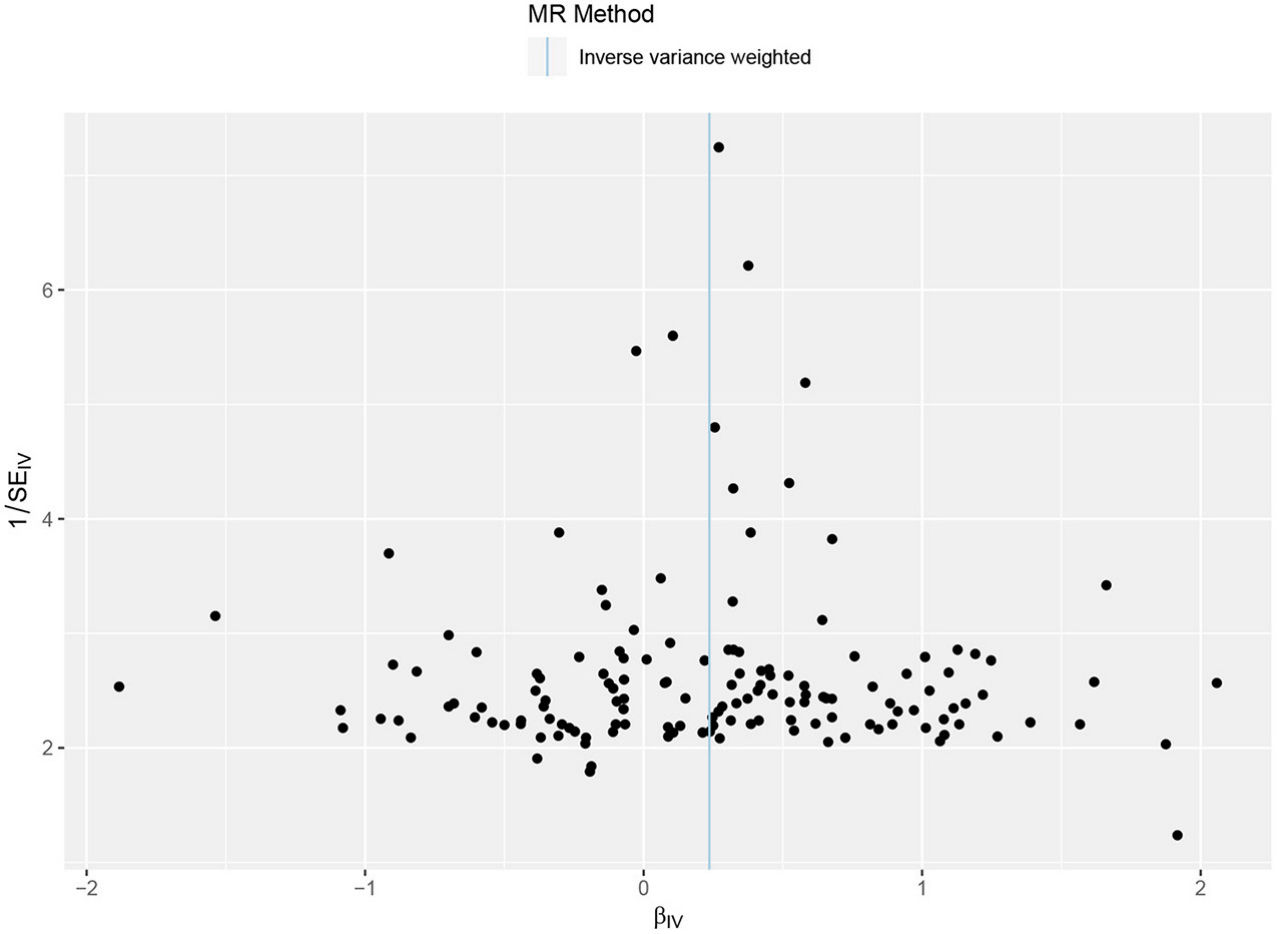
Funnel plot of the Mendelian randomization analysis. The X axis presents the estimate of the causal effect, and the Y axis presents the relevant inverse standard error. The dots indicate each SNP, and the line indicates the overall estimate using IVW method. SNP, single-nucleotide polymorphism; IVW, inverse variance-weighted.

The primary result using IVW method is shown in Figure 2, Supplementary Figure 1. The OR and 95% CI of AF per SD increased BW were 1.27 (1.14–1.40), *p* = 5.70 × 10^−6^. This result suggested that the genetically predicted BW was causally associated with the risk of AF. The power of the primary analysis was 1.00, as shown in Supplementary Table 4. In addition, the visual inspection of the association of each SNP with BW and its effect on AF is shown in Supplementary Figure 2. The results of sensitivity analyses with various statistical methods were consistent with our primary result, as shown in Figure 2. And the leave-one-out analysis suggested that the overall estimate was unlikely driven by any single SNP, as shown in Supplementary Figure 3. The result of MR-Egger intercept test is shown in Table 1. The intercept and 95% CI were −0.004 (−0.010, 0.003), *p* = 0.24, which suggested no evidence of directional pleiotropy. Additional evidence against directional pleiotropy was provided by the symmetric funnel plot, as shown in Figure 3.

**Table 1:** MR-Egger intercept test of the causal association between birth weight and atrial fibrillation.

**Exposure**	**Outcome**	**Intercept (95% CI)**	***p***
Birth weight	Atrial fibrillation	−0.004 (−0.010, 0.003)	0.24

*CI, confidence interval*.

### BMI Partially Mediated the Association Between BW and AF

**Figure 4:**
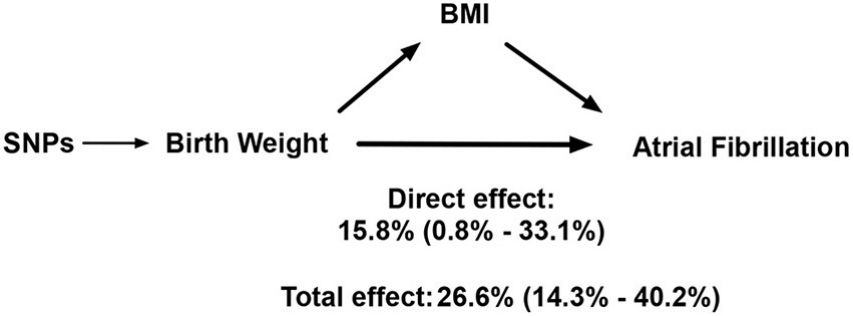
A model for how birth weight impacts atrial fibrillation. SNP, single-nucleotide polymorphism; BMI, body mass index.

As shown in Supplementary Figure 4, genetically predicted BW was robustly associated with elevated BMI. Table 2 presents the effect of BW on AF when adjusted for BMI. The OR and 95% CI of AF per SD increased BW conditional on BMI were 1.16 (1.01–1.33), *p* = 0.04. A model of mediation analysis is presented in Figure 4. The total effect of elevated BW was to increase the risk of AF by 26.6% (14.3–40.2%), while the direct effect of elevated BW conditional on BMI accounted for an increase of AF risk by 15.8% (0.8–33.1%). This indicated that BMI was partially involved in the association between BW and AF, while BW was still an independent risk factor for AF.

**Table 2:** Multivariable Mendelian randomization analysis for association between birth weight and atrial fibrillation conditional on body mass index.

**Exposure**	**Outcome**	**OR (95% CI)**	***P***
Birth weight	Atrial fibrillation	1.16 (1.01, 1.33)	0.04
Body mass index	Atrial fibrillation	1.70 (1.02, 2.84)	0.04

*OR, odds ratio; CI, confidence interval*.

## Discussion

This MR study showed that genetically elevated BW was significantly associated with the increased risk of AF in later life. Though BMI may partially mediate this association, BW is still a robust risk factor for AF.

The association between BW and the risk of cardiovascular diseases was first described in 1989 (9). Since then, multiple studies have confirmed an established risk of low weight at birth on cardiovascular diseases in adulthood, such as coronary artery disease, myocardial infarction, type 2 diabetes mellitus, and hypertension (9–13). However, only four previous studies have assessed the potential association between BW and the risk of AF, with conflicting results (14–17). The Women's Health Study of 27,982 women, including 735 AF cases during a median follow-up of 14.5 years, indicated that BW was significantly associated with the incident AF among women (14). The Atherosclerosis Risk in Communities cohort of 10,132 individuals, identifying 882 AF cases during an average follow-up of 10.3 years, demonstrated that low BW was independently associated with increased risk of AF (15). The prospective cohorts in Sweden of 29,551 men and 23,454 women, comprising 2,711 men and 1,491 women who developed AF during 12 years of follow-up, showed that low BW in men and high BW both in men and women were associated with higher risk of AF (16). The Helsinki Birth Cohort Study of 13,345 individuals, including 907 incident cases during 70.5 years of follow-up, suggested a significant U-shaped association between BW and AF (17). Although several recognized confounding factors have been adjusted in these studies, the influence of unmeasured confounders was inevitable, which may account for the divergent results.

MR analysis can avoid the potential unmeasured confounders thus making stronger causal inference (19, 20). Previously, a large-scale MR study (12) supported that lower BW was causally associated with an increased risk of coronary artery disease, myocardial infarction, and type 2 diabetes mellitus, which were well-recognized risk factors for AF (7, 37). This seems to be diametrically opposed to the results we present here, the underlying mechanism of which is unknown. The present study provided evidence for the causal association between the elevated BW and the increased risk of AF. Though BMI may partially mediate this association, BW is still a robust risk factor for AF. Increased atrial size, involved in the pathophysiology of AF, has been recognized as a well-established risk factor for AF (38). Given the potential association between body mass and left atrial size, it is plausible that the association of BW with AF may be mediated through body mass and left atrial size subsequently (39). The strong association between BMI and diastolic dysfunction is another possible explanation for the relationship between BW and AF mediated by BMI (40, 41). Further investigations are warranted to explore the exact mechanism by which high BW is associated with high AF risk.

A major strength of this study is the design of MR analysis, which can avoid the potential unmeasured confounders and reverse causation in comparison to conventional observational studies (19, 20). The use of MR analysis enables a causal inference for the association between BW and the risk of AF. Another important strength is that the genetic data were derived from the largest GWAS to date. An extremely large sample size ensures good statistical power for MR analysis. An additional strength is that large number of SNPs were identified in this study, which is more conducive to reliable and precise results. In addition, our findings plausibly suggest a feasible and effective prevention to ease the burden of AF by preventing the overweight of newborns. A balanced diet and regular check-ups during pregnancy are required to control the BW of newborns, which is of great significance to the prevention of AF. People born with high weight can pay more attention to their risk of developing AF and adopt a healthy lifestyle or other way to lower their risk.

There are several limitations in our study that also require discussion. First, a potential threat to the reliability of the results is the violation of the requisite MR assumptions. Potential pleiotropy could not be completely ruled out, which may lead to biased estimates. However, no evidence of pleiotropic effect was observed in the pleiotropy assessment and sensitivity analyses with robust methods. Second, there was partial overlap between the individuals included in the GWAS datasets for BW and AF, which could bias the result if substantial. Although the precise degree of the overlap was difficult to quantify, the UK biobank, one of the three components of BW GWAS, was also part of the AF GWAS (12.9%). The real proportion was probably smaller; thus, the risk of bias from sample overlap was likely to be low. Third, most of the individuals in our study were of European ancestry, which may limit the generalizability of the results to other population. Fourth, we were unable to address the sexual disparities in association between BW and AF risk because sex-specific genetic data were not available. Fifth, although the phenotypes of multiple births and gestational age <37 weeks have been almost excluded, a non-linear association between BW and AF could also exist, which might bias the result. Finally, we only revealed the causal association between BW and the risk of AF from a genetic perspective, without involving the maternal intrauterine environment.

## Conclusions

This study indicated that elevated BW was significantly associated with increased lifelong risk of AF, which may be partially mediated by BMI.

## Data Availability Statement

The datasets presented in this study can be found in online repositories. The names of the repository/repositories and accession number(s) can be found in the article/Supplementary Material.

## Author Contributions

SC, TX, and WZ designed the study and wrote the analysis plan. SC and FY undertook analyses. SC wrote the first draft of the manuscript with critical revisions from TX, YW, and GF. All authors interpreted the results in the study and gave final approval of the version to be published.

## Conflict of Interest

The authors declare that the research was conducted in the absence of any commercial or financial relationships that could be construed as a potential conflict of interest.

## Publisher's Note

All claims expressed in this article are solely those of the authors and do not necessarily represent those of their affiliated organizations, or those of the publisher, the editors and the reviewers. Any product that may be evaluated in this article, or claim that may be made by its manufacturer, is not guaranteed or endorsed by the publisher.

## References

[1] January CT Wann LS Alpert JS Calkins H Cigarroa JE Cleveland JC Jr. 2014 AHA/ACC/HRS guideline for the management of patients with atrial fibrillation: a report of the American College of Cardiology/American Heart Association Task Force on practice guidelines and the Heart Rhythm Society. Circulation. (2014) 130:e199–267. 10.1016/j.jacc.2014.03.022 24682347 PMC4676081

[2] Kirchhof P Benussi S Kotecha D Ahlsson A Atar D Casadei B. 2016 ESC guidelines for the management of atrial fibrillation developed in collaboration with EACTS. Eur Heart J. (2016) 37:2893–962. 10.15829/1560-4071-2017-7-7-86 27567408

[3] Chugh SS Havmoeller R Narayanan K Singh D Rienstra M Benjamin EJ. Worldwide epidemiology of atrial fibrillation: a Global Burden of Disease 2010. Study. Circul. (2014) 129:837–47. 10.1161/CIRCULATIONAHA.113.005119 24345399 PMC4151302

[4] Zulkifly H Lip GYH Lane DA. Epidemiology of atrial fibrillation. Int J Clin Pract. (2018) 72:e13070. 10.1111/ijcp.13070 29493854

[5] Rosenfeld LE Amin AN Hsu JC Oxner A Hills MT Frankel DS. The Heart Rhythm Society/American college of physicians atrial fibrillation screening and education initiative. Heart Rhythm. (2019) 16:e59–e65. 10.1016/j.hrthm.2019.04.007 30954599

[6] Groshans KA Leary MC. Management of atrial fibrillation. In: Caplan LR, Biller J, Leary M, Lo E, Thomas A, Yenari M, Zhang J, editors. Primer on Cerebrovascular Diseases, Second Edition. Academic Press (2017). p. 759–66. 10.1016/B978-0-12-803058-5.00146-6

[7] Staerk L Sherer JA Ko D Benjamin EJ Helm RH. Atrial fibrillation: epidemiology, pathophysiology, and clinical outcomes. Circ Res. (2017) 120:1501–17. 10.1161/CIRCRESAHA.117.309732 28450367 PMC5500874

[8] Ferrari R Bertini M Blomstrom-Lundqvist C Dobrev D Kirchhof P Pappone C. An update on atrial fibrillation in 2014: from pathophysiology to treatment. Int J Cardiol. (2016) 203:22–9. 10.1016/j.ijcard.2015.10.089 26490502

[9] Harris MTM Berstein L. Weight in infancy and death from ischaemic heart disease. Lancet. (1989). 2:1335. 10.1016/S0140-6736(89)91939-9 2574280

[10] Horikoshi M Beaumont RN Day FR Warrington NM Kooijman MN Fernandez-Tajes J. Genome-wide associations for birth weight and correlations with adult disease. Nature. (2016) 538:248–52. 10.1038/nature19806 27680694 PMC5164934

[11] Tian J Qiu M Li Y Zhang X Wang H Sun S. Contribution of birth weight and adult waist circumference to cardiovascular disease risk in a longitudinal study. Sci Rep. (2017) 7:9768. 10.1038/s41598-017-10176-6 28852140 PMC5575020

[12] Zeng P Zhou X. Causal association between birth weight and adult diseases: evidence from a mendelian randomization analysis. Front Genet. (2019) 10:618. 10.3389/fgene.2019.00618 31354785 PMC6635582

[13] Knop MR Geng TT Gorny AW Ding R Li C Ley SH. Birth weight and risk of type 2 diabetes mellitus, cardiovascular disease, and hypertension in adults: a meta-analysis of 7 646 267 participants from 135 studies. J Am Heart Assoc. (2018) 7:e008870. 10.1161/JAHA.118.008870 30486715 PMC6405546

[14] Conen D Tedrow UB Cook NR Buring JE Albert CM. Birth weight is a significant risk factor for incident atrial fibrillation. Circulation. (2010) 122:764–70. 10.1161/CIRCULATIONAHA.110.947978 20697028 PMC2927709

[15] Lawani SO Demerath EW Lopez FL Soliman EZ Huxley RR Rose KM. Birth weight and the risk of atrial fibrillation in whites and African Americans: the Atherosclerosis Risk In Communities (ARIC) study. BMC Cardiovasc Disord. (2014) 14:69. 10.1186/1471-2261-14-69 24885251 PMC4045869

[16] Larsson SC Drca N Jensen-Urstad M Wolk A. Incidence of atrial fibrillation in relation to birth weight and preterm birth. Int J Cardiol. (2015) 178:149–52. 10.1016/j.ijcard.2014.10.138 25464240

[17] Johnson LSB Salonen M Kajantie E Conen D Healey JS Osmond C. Early life risk factors for incident atrial fibrillation in the helsinki birth cohort study. J Am Heart Assoc. (2017) 6:e006036. 10.1161/JAHA.117.006036 28649086 PMC5669198

[18] Frost L Olsen J. Birth weight and atrial fibrillation: a causal link? Circulation. (2010) 122:759–60. 10.1161/CIRCULATIONAHA.110.970632 20697025

[19] Sekula P Del Greco MF Pattaro C Kottgen A. Mendelian randomization as an approach to assess causality using observational data. J Am Soc Nephrol. (2016) 27:3253–65. 10.1681/ASN.2016010098 27486138 PMC5084898

[20] Holmes MV Ala-Korpela M Smith GD. Mendelian randomization in cardiometabolic disease: challenges in evaluating causality. Nat Rev Cardiol. (2017) 14:577–90. 10.1038/nrcardio.2017.78 28569269 PMC5600813

[21] Ference BA Julius S Mahajan N Levy PD Williams KA Sr Flack JM. Clinical effect of naturally random allocation to lower systolic blood pressure beginning before the development of hypertension. Hypertension. (2014) 63:1182–8. 10.1161/HYPERTENSIONAHA.113.02734 24591335

[22] Pierce BL Burgess S. Efficient design for Mendelian randomization studies: subsample and 2-sample instrumental variable estimators. Am J Epidemiol. (2013) 178:1177–84. 10.1093/aje/kwt084 23863760 PMC3783091

[23] Lawlor DA. Commentary: two-sample Mendelian randomization: opportunities and challenges. Int J Epidemiol. (2016) 45:908–15. 10.1093/ije/dyw127 27427429 PMC5005949

[24] Warrington NM Beaumont RN Horikoshi M Day FR Helgeland O Laurin C. Maternal and fetal genetic effects on birth weight and their relevance to cardio-metabolic risk factors. Nat Genet. (2019) 51:804–14. 10.1038/s41588-019-0403-1 31043758 PMC6522365

[25] Nielsen JB Thorolfsdottir RB Fritsche LG Zhou W Skov MW Graham SE. Biobank-driven genomic discovery yields new insight into atrial fibrillation biology. Nat Genet. (2018) 50:1234–9. 10.1038/s41588-018-0171-3 30061737 PMC6530775

[26] Machiela MJ Chanock SJ. LDlink: a web-based application for exploring population-specific haplotype structure and linking correlated alleles of possible functional variants. Bioinformatics. (2015) 31:3555–7. 10.1093/bioinformatics/btv402 26139635 PMC4626747

[27] Johnson AD Handsaker RE Pulit SL Nizzari MM O'Donnell CJ de Bakker PI. SNAP: a web-based tool for identification and annotation of proxy SNPs using HapMap. Bioinformatics. (2008) 24:2938–9. 10.1093/bioinformatics/btn564 18974171 PMC2720775

[28] Johnson T. Efficient calculation for multi-snp genetic risk scores. In: ASHG Annual Meeting. San Francisco, CA (2012).

[29] Freeman G Cowling BJ Schooling CM. Power and sample size calculations for Mendelian randomization studies using one genetic instrument. Int J Epidemiol. (2013) 42:1157–63. 10.1093/ije/dyt110 23934314

[30] Bowden J Davey Smith G Haycock PC Burgess S. Consistent estimation in mendelian randomization with some invalid instruments using a weighted median estimator. Genet Epidemiol. (2016) 40:304–14. 10.1002/gepi.21965 27061298 PMC4849733

[31] Bowden J Davey Smith G Burgess S. Mendelian randomization with invalid instruments: effect estimation and bias detection through Egger regression. Int J Epidemiol. (2015) 44:512–25. 10.1093/ije/dyv080 26050253 PMC4469799

[32] Verbanck M Chen CY Neale B Do R. Detection of widespread horizontal pleiotropy in causal relationships inferred from Mendelian randomization between complex traits and diseases. Nat Genet. (2018) 50:693–8. 10.1038/s41588-018-0099-7 29686387 PMC6083837

[33] Sterne JA Sutton AJ Ioannidis JP Terrin N Jones DR Lau J. Recommendations for examining and interpreting funnel plot asymmetry in meta-analyses of randomised controlled trials. BMJ. (2011) 343:d4002. 10.1136/bmj.d4002 21784880

[34] Yengo L Sidorenko J Kemper KE Zheng Z Wood AR Weedon MN. Meta-analysis of genome-wide association studies for height and body mass index in approximately 700000 individuals of European ancestry. Hum Mol Genet. (2018) 27:3641–9. 10.1093/hmg/ddy271 30124842 PMC6488973

[35] Yavorska OO Burgess S. Mendelian Randomization: an R package for performing Mendelian randomization analyses using summarized data. Int J Epidemiol. (2017) 46:1734–9. 10.1093/ije/dyx034 28398548 PMC5510723

[36] Hemani G Zheng J Elsworth B Wade KH Haberland V Baird D. The MR-Base platform supports systematic causal inference across the human phenome. Elife. (2018) 7:e34408. 10.7554/eLife.34408 29846171 PMC5976434

[37] Psaty BM Manolio TA Kuller LH Kronmal RA Cushman M Fried LP. Incidence of and risk factors for atrial fibrillation in older adults. Circulation. (1997) 96:2455–61. 10.1161/01.CIR.96.7.2455 9337224

[38] Abhayaratna WP Seward JB Appleton CP Douglas PS Oh JK Tajik AJ. Left atrial size: physiologic determinants and clinical applications. J Am Coll Cardiol. (2006) 47:2357–63. 10.1016/j.jacc.2006.02.048 16781359

[39] Armstrong AC Gidding SS Colangelo LA Kishi S Liu K Sidney S. Association of early adult modifiable cardiovascular risk factors with left atrial size over a 20-year follow-up period: the CARDIA study. BMJ Open. (2014) 4:e004001. 10.1136/bmjopen-2013-004001 24384901 PMC3902509

[40] Berkovitch A Kivity S Klempfner R Segev S Milwidsky A Erez A. Body mass index and the risk of new-onset atrial fibrillation in middle-aged adults. Am Heart J. (2016) 173:41–8. 10.1016/j.ahj.2015.11.016 26920595

[41] Powell BD Redfield MM Bybee KA Freeman WK Rihal CS. Association of obesity with left ventricular remodeling and diastolic dysfunction in patients without coronary artery disease. Am J Cardiol. (2006) 98:116–20. 10.1016/j.amjcard.2006.01.063 16784933

