# Supplementary material for: Genetic Determinants of Increased Body Mass Index Partially Mediate the Effect of Elevated Birth Weight on the Increased Risk of Atrial Fibrillation
Source: Front Cardiovasc Med. 2021 Aug 6;8:701549. doi: 10.3389/fcvm.2021.701549 (PMC8377229; doi:10.3389/fcvm.2021.701549)
Supplement: Supplementary file 1 [file Data_Sheet_1.docx]

**SUPPLEMENTAL MATERIAL**

**Table S1.** Overview of studies contributing to genetic associations with birth weight.^1^

| Study | Ancestry group | Country of origin | Year(s) of birth | Sample size (M/F) | Data collection | Phenotype exclusions | Mean (SD) birth weight (grams) | Median (IQR) GA (week) at delivery |
| --- | --- | --- | --- | --- | --- | --- | --- | --- |
| 1958 British Birth Cohort | European | UK | 1958 | 4,595 (2,320/2,275) | Measured by midwives; supplemented with obstetric records and interviews with mothers | Multiple births, GA <37 weeks | 3359 (483) | 40 (39-41) |
| ABCD | European | Netherlands | 2003-2004 | 1,107 (536/571) | Youth Health Care Registration | Multiple births, GA <37 weeks | 3555 (479) | 40 (39-41) |
| ALSPAC | European | UK | ~1992 | 7,285 (3,722/3,563) | Identified from obstetric data, records from the ALSPAC measurers, and birth notification | Multiple births, GA <37 weeks, 5 SD winsorisation | 3490 (476) | 40 (40-41) |
| CHOP-Caucasian | European | USA | 1988-present | 9,405 (5,040/4,365) | Questionnaire and medical records | Multiple births, GA <37 weeks (when available) | 3398 (569) | N/A |
| CoLaus | European | Switzerland | 1928-1970 | 2,089 (892/1,197) | Self-reported as adults | N/A | 3352 (675) | N/A |
| COPSAC-2000 | European | Denmark | 1998-2001 | 352 (173/179) | Medical records | Multiple births, GA <37 weeks | 3555 (485) | 40 (39-41) |
| COPSAC-2010 | European | Denmark | 2008-2011 | 589 (306/283) | Medical records | Multiple births, GA <37 weeks | 3588 (481) | 40 (39-41) |
| COPSAC- REGISTRY | European | Denmark | 1987-1999 | 1,210 (804/406) | Medical Records | Multiple births, GA <37 weeks | 3553 (488) | 40 (39-41) |
| DNBC | European | Denmark | 1996-2003 | 915 (475/440) | Danish Medical Birth Register | Multiple births, GA <37 weeks, congenital abnormalities | 3699 (468) | 40 (40-41) |
| ERF | European | Netherlands | Various | 459 (187/272) | Interview | GA <37 weeks | 3039 (644) | N/A |
| EPIC | European | UK | 1993-1997 | 8,939 (3,448/5,491) | Self-reported | N/A | 3358 (772) | N/A |
| Fenland (GA+) | European | UK | 1950-1975 | 5,188 (2,088/3,100) | Self-reported as adults | GA described as "very pre-term" or "pre-term" | 3394 (555) | N/A |
| Fenland (GA-) | European | UK | 1950-1975 | 833 (509/324) | Self-reported as adults | None | 3354 (624) | N/A |
| Generation R | European | Netherlands | 2002-2006 | 2,701 (1,378/1,323) | Hospital records and community midwives | Multiple births, GA <37 weeks | 3574 (488) | 40 (39-41) |
| GINIplus & LISA (GA+) | European | Germany | 1996-1999 | 656 (360/296) | Parental report of medical records | Multiple births, GA <37 weeks, <2500g | 3443 (415) | 40 (39-41) |
| GINIplus & LISA (GA-) | European | Germany | 1996-1999 | 790 (391/399) | Parental report of medical records | Multiple births, GA <37 weeks, <2500g | 3423 (433) | N/A |
| GOYA | European | Denmark | 1943-1952 | 149/0 (obese), 141/0 (control) | School health records | N/A | 3553 (711) | N/A |
| GOYA offsprings | European | Denmark | 1996-2002 | 907 (461/446) | Measured by midwives and obtained from the Danish National Birth Registry | Multiple births, GA <37 weeks | 3753 (496) | 40 (39-41) |
| HAPO-EUR | Caucasian | Canada, UK, Australia | 2000-2006 | 1,333 (659/664) | Measured within 72 hours of birth using methods and equipment standardized across all centres | Multiple births, GA <37 weeks, abs (BW- z)>5 | 3425 (498) | 40 (39-41) |
| HBCS | European | Finland | 1934-1944 | 1,472 (639/833) | Birth records | Multiple births, GA <37 weeks | 3444 (454) | 40 (39-41) |
| HEALTH2006 | European | Denmark | 1927-1988 | 1,176 (442/734) | Self-reported as adults | None | 3343 (593) | NA |
| INMA | European | Spain | 1997-2006 | 1,021 (527/494) | Well-trained midwives and nurses | None | 3278 (423) | 40 (39-41) |
| INTER99 | European | Denmark | 1939-1969 | 4,243 (1,981/2,262) | Measured by midwives and obtained from obstetric record registry | Multiple births, GA <37 weeks | 3433 (485) | N/A |
| Leipzig | European | Germany | 1985 - 2010 | 597 (304/293) | Questionnaire to mothers, documentation of medical screening examination if available | GA <37 weeks | 3527 (536) | 40 (39-40) |
| NEO | European | Netherlands | 1943-1963 | 504 (exact; 200/304), 3,215 (range; 1,450/1,765) | Questionnaire | N/A | 3514 (1271) | N/A |
| NFBC1966 | European | Finland | 1966 | 5,009 (2,393/2,616) | Measured in hospitals | Multiple births, GA <37 weeks or unknown | 3541 (489) | 40 (39-41) |
| NFBC1986 | European | Finland | 1986 | 4,680 (2,306/2,374) | Measured in hospitals | Multiple births, GA <37 weeks or unknown | 3572 (535) | 40 (39-40) |
| NTR | European | Netherlands | 1926-1998 | 1,265 (447/818) | Parental report or self-reported | Multiple births, GA <37 weeks | 3343 (601) | 40 (40-40) |
| ORCADES | European | Scotland | 1920-1991 | 960 (330/630) | Self-reported as adults | N/A | 3488 (640) | N/A |
| PANIC | European | Finland | 1999-2002 | 436 (231/205) | Medical records and parental questionnaire | Multiple births, GA <37 weeks | 3588 (474) | 40 (39-41) |
| RAINE | European | Australia | 1989-1991 | 1,347 (693/654) | Recorded at delivery by study personnel or obtained from hospital reports | Multiple births, GA <37 weeks | 3449 (470) | 40 (39-41) |
| SKOT | European | Denmark | 2006-2007, 2011-2013 | 348 (173/175) | Measured by midwives and general practitioners; obtained from health records kept by the parents | Multiple births, GA <37 weeks | 3607 (483) | 40 (39-41) |
| SORBS | European | Germany | 1925-1988 | 298 (113/185) | Interview at recruitment | N/A | 3393 (673) | N/A |
| STRIP | European | Finland | 1989-1991 | 599 (311/288) | Medical records | Multiple births, GA <37 weeks | 3619 (465) | 40 (39-40) |
| TEENAGE (GA+) | European | Greece | 1993-1998 | 279 (126/153) | Measured by midwives or paediatricians; supplemented with data from mothers’ interviews | GA <37 weeks | 3336 (445) | 40 (38-40) |
| TEENAGE (GA-) | European | Greece | 1993-1998 | 551 (234/317) | Measured by midwives or paediatricians; supplemented with data from mothers’ interviews | N/A | 3341 (449) | N/A |
| TDCOB-cases | European | Denmark | 1987-2007 | 669 (391/278) | Measured by midwives and registered in Danish Civil Registry | Multiple births | 3660 (540) | 40 (39-41) |
| TDCOB-controls | European | Denmark | 1991-2006 | 560 (211/349) | Measured by midwives and registered in Danish Civil Registry | Multiple births | 3540 (502) | 40 (39-41) |
| YFS | European | Finland | 1962-1977 | 1,915 (861/1,054) | Mothers’ interview | Multiple births, GA >3 weeks pre-term | 3572 (475) | N/A |
| CHOP-AA | African American | USA | 1988-present | 6,635 (3,343/3,292) | Questionnaire and medical records | Multiple births, GA <37 weeks (when available) | 3231 (546) | N/A |
| CLHNS | Filipino | Philippines | 1983-1984 | 1,449 (755/694) | Local birth attendants | Multiple births, GA <37 weeks | 3043 (403) | 40 (38-40) |
| Generation R Turkish | Turkish | Netherlands | 2002-2006 | 420 (215/205) | Hospital records and community midwives | Multiple births, GA <37 weeks | 3424 (463) | 40 (39-41) |
| Generation R Moroccan | Moroccan | Netherlands | 2002-2006 | 365 (188/177) | Hospital records and community midwives | Multiple births, GA <37 weeks | 3533 (416) | 41 (40-41) |
| Generation R Surinamese | Surinamese | Netherlands | 2002-2006 | 395 (215/180) | Hospital records and community midwives | Multiple births, GA <37 weeks | 3216 (532) | 40 (39-41) |
| HAPO-AC | Afro- Caribbean | Barbados | 2000-2006 | 1,052 (544/508) | Measured within 72 hours of birth using methods and equipment standardized across all centres | Multiple births, GA <37 weeks, abs(BW- z)>5 | 3228 (441) | 40 (39-41) |
| HAPO-MA | Hispanic | USA | 2000-2006 | 612 (303/309) | Measured within 72 hours of birth using methods and equipment standardized across all centres | Multiple births, GA <37 weeks, abs(BW- z)>5 | 3435 (435) | 40 (39-41) |
| HAPO-TH | Thai | Thailand | 2000-2006 | 1,180 (575/605) | Measured within 72 hours of birth using methods and equipment standardized across all centres | Multiple births, GA <37 weeks, abs(BW- z)>5 | 3093 (382) | 40 (39-41) |
| SCORM | Chinese | Singapore | 1992-1995 | 840 (420/420) | Documented medical record booklet | GA <37 weeks | 3205 (450) | 39 (38-40) |
| UK Biobank | All | UK | 2006-2010 | 227,530 (89,037/138,493) | Self-reported as adults | Multiple births, birth weight <2500g or >4500g | 3387 (419) | N/A |

M, Males; F, Females; SD, standard deviation; IQR, interquartile range; GA, gestational age; N/A, not applicable.

**Table S2.** Overview of studies contributing to genetic associations with atrial fibrillation.^2^

| Study | | Ancestry group | Sample size | Cases | Controls |
| --- | --- | --- | --- | --- | --- |
| The Nord-Trøndelag Health Study (HUNT) | | European | 69,635 | 6,493 | 63,142 |
| deCODE | | European | 371,632 | 13,471 | 358,161 |
| the Michigan Genomics Initiative (MGI) | | European | 12,275 | 1,226 | 11,049 |
| DiscovEHR | | European | 48,482 | 6,679 | 41,803 |
| UK Biobank | | European | 395,739 | 14,820 | 380,919 |
| AFGen Consortium (35 cohorts) | Atrial Fibrillation Biobank LMU/Cooperative Health Research in the Region of Augsburg (AFLMU/KORA) | European | 886 | 448 | 438 |
|  | Age, Gene/Environment Susceptibility Study (AGES) Reykjavik study | European | 2893 | 785 | 2108 |
|  | Angiography and Genes Study (ANGES) | European | 779 | 226 | 553 |
|  | Atherosclerosis Risk in Communities Study (ARIC) | African American | 2768 | 278 | 2490 |
|  | Atherosclerosis Risk in Communities Study (ARIC) | European | 8880 | 1420 | 7460 |
|  | Basel Atrial Fibrillation Cohort Study (BEAT-AF) | European | 3040 | 1520 | 1520 |
|  | Biobank Japan | East Asian | 4130 | 837 | 3293 |
|  | Cleveland Clinic Lone Atrial Fibrillation GeneBank Study (CCAF) | European | 3536 | 606 | 2930 |
|  | Cardiovascular Health Study (CHS) | African American | 801 | 189 | 612 |
|  | Cardiovascular Health Study (CHS) | European | 3201 | 1011 | 2190 |
|  | COROGENE | European | 2226 | 248 | 1978 |
|  | Framingham Heart Study (FHS) | European | 4404 | 880 | 3524 |
|  | Finnish Cardiovascular Study (FINCAVAS) | European | 2879 | 971 | 1908 |
|  | Generation Scotland: Scottish Family Health Study (GS:SFHS) | European | 6854 | 203 | 6651 |
|  | Ludwigshafen Risk and Cardiovascular Health (LURIC) | European | 3034 | 368 | 2666 |
|  | Malmö Diet and Cancer study (MDCS) | European | 5877 | 1232 | 4645 |
|  | Mount Sinai BioMe Biobank | African American | 2306 | 174 | 2132 |
|  | Mount Sinai BioMe Biobank | European | 1151 | 291 | 860 |
|  | Mount Sinai BioMe Biobank | Hispanic | 3358 | 277 | 3081 |
|  | Multi-Ethnic Study of Atherosclerosis (MESA) | European | 2527 | 155 | 2372 |
|  | Massachusetts General Hospital Atrial Fibrillation study (MGH AF) | European | 1277 | 366 | 911 |
|  | Massachusetts General Hospital Cardiology and Metabolic Patient cohort (MGH CAMP) | European | 2793 | 665 | 2128 |
|  | Prospective Investigation of Vasculature in Uppsala Seniors (PIVUS) | European | 949 | 154 | 795 |
|  | Prevention of Renal and Vascular Endstage Disease (PREVEND) | European | 3520 | 113 | 3407 |
|  | PROspective Study of Pravastatin in the Elderly at Risk (PROSPER) | European | 5244 | 505 | 4739 |
|  | Rotterdam Study I (RS I) | European | 5947 | 1025 | 4922 |
|  | Rotterdam Study II (RS II) | European | 1806 | 146 | 1660 |
|  | Rotterdam Study III (RS III) | European | 3030 | 121 | 2909 |
|  | Study of Health in Pomerania (SHIP) | European | 1921 | 106 | 1815 |
|  | Sao Paolo Heart Failure Cohort (SPHFC) | Brazilian | 955 | 197 | 758 |
|  | Swedish Twin Registry (TWINGENE) | European | 6813 | 403 | 6410 |
|  | Uppsala Longitudinal Study of Adult Men (ULSAM) | European | 1120 | 294 | 826 |
|  | Vanderbilt University Medical Center BioVU Biorepository | European | 10185 | 428 | 9757 |
|  | Wellcome Trust Case Control Consortium 2 Munich (WTCCC2-Munich) | European | 1127 | 330 | 797 |
|  | Women’s Genome Health Study (WGHS) | European | 20856 | 959 | 19897 |

**Table S3.** The characteristics of 144 SNPs and their genetic associations with the birth weight and the atrial fibrillation.^1,2^

| SNP | Chr | Pos | EA | OA | EAF | F | Birth Weight | | Atrial Fibrillation | |
| --- | --- | --- | --- | --- | --- | --- | --- | --- | --- | --- |
|  |  |  |  |  |  |  | Beta | SE | Beta | SE |
| rs10147938 | 14 | 31885951 | T | C | 0.387 | 34 | 0.015 | 0.003 | -0.0015 | 0.0068 |
| rs10181515 | 2 | 227019461 | T | C | 0.225 | 49 | 0.021 | 0.003 | 0.023 | 0.0079 |
| rs10221267 | 17 | 68464662 | T | C | 0.512 | 46 | 0.017 | 0.003 | 0.0275 | 0.0066 |
| rs10265057 | 7 | 47275737 | G | A | 0.092 | 39 | 0.027 | 0.004 | 0.009 | 0.0113 |
| rs10283100 | 8 | 120596023 | G | A | 0.946 | 58 | 0.042 | 0.006 | 0.0092 | 0.0152 |
| rs10495563 | 2 | 9662210 | A | G | 0.664 | 69 | 0.022 | 0.003 | 0.0248 | 0.0077 |
| rs10883846 | 10 | 104958244 | C | T | 0.615 | 44 | 0.017 | 0.003 | 0.0207 | 0.0069 |
| rs10913200 | 1 | 176521655 | G | A | 0.972 | 45 | 0.051 | 0.008 | 0.0108 | 0.0239 |
| rs10935733 | 3 | 148622968 | T | C | 0.399 | 56 | 0.019 | 0.003 | -0.0044 | 0.0068 |
| rs11042596 | 11 | 2118860 | T | G | 0.336 | 105 | 0.027 | 0.003 | -0.0247 | 0.0073 |
| rs11082304 | 18 | 20720973 | T | G | 0.508 | 41 | 0.016 | 0.003 | 0.0185 | 0.0067 |
| rs11085720 | 19 | 10317763 | A | G | 0.438 | 36 | 0.015 | 0.003 | 0.0134 | 0.0068 |
| rs1112718 | 10 | 94479107 | G | A | 0.404 | 105 | 0.026 | 0.003 | 0.0176 | 0.0068 |
| rs112139215 | 7 | 73034559 | A | C | 0.068 | 90 | 0.047 | 0.005 | 0.0029 | 0.0135 |
| rs1129156 | 19 | 40719076 | T | C | 0.268 | 36 | 0.017 | 0.003 | 0.0042 | 0.0075 |
| rs116276359 | 1 | 151821430 | A | C | 0.031 | 36 | 0.043 | 0.008 | -0.009 | 0.0211 |
| rs11646700 | 16 | 68421668 | G | A | 0.495 | 36 | 0.015 | 0.003 | -0.0066 | 0.0067 |
| rs116807401 | 4 | 135121721 | C | T | 0.018 | 67 | 0.077 | 0.01 | 0.0323 | 0.0302 |
| rs11704481 | 22 | 45732328 | G | A | 0.403 | 35 | 0.015 | 0.003 | -0.0162 | 0.0069 |
| rs11708067 | 3 | 123065778 | G | A | 0.238 | 196 | 0.041 | 0.003 | 0.0238 | 0.0079 |
| rs11711420 | 3 | 183349010 | T | G | 0.747 | 44 | 0.019 | 0.003 | 0.0195 | 0.0076 |
| rs118106744 | 12 | 21936398 | C | T | 0.948 | 34 | 0.033 | 0.006 | 0.0239 | 0.0158 |
| rs11867479 | 17 | 68090207 | T | C | 0.353 | 42 | 0.017 | 0.003 | -0.0012 | 0.007 |
| rs11983722 | 7 | 46298647 | A | T | 0.938 | 38 | 0.032 | 0.005 | -0.0031 | 0.0133 |
| rs1203876 | 20 | 22540915 | C | A | 0.046 | 41 | 0.038 | 0.006 | 0.0176 | 0.0154 |
| rs12104672 | 2 | 109151173 | T | G | 0.574 | 31 | 0.014 | 0.003 | 0.0149 | 0.0068 |
| rs12153596 | 5 | 158410178 | C | T | 0.619 | 34 | 0.015 | 0.003 | -0.0044 | 0.0068 |
| rs12401656 | 1 | 43456767 | G | A | 0.865 | 47 | 0.025 | 0.004 | 0.0079 | 0.0098 |
| rs1242516 | 17 | 17387079 | C | T | 0.86 | 34 | 0.021 | 0.004 | 0.005 | 0.0098 |
| rs12656216 | 5 | 36160668 | A | G | 0.788 | 35 | 0.018 | 0.003 | 0.025 | 0.0081 |
| rs12802960 | 11 | 58174775 | C | T | 0.211 | 31 | 0.017 | 0.003 | 0.0015 | 0.0081 |
| rs12896104 | 14 | 74306014 | G | A | 0.674 | 32 | 0.015 | 0.003 | 0.0041 | 0.0072 |
| rs13231367 | 7 | 127509070 | G | A | 0.714 | 38 | 0.017 | 0.003 | -0.0185 | 0.0073 |
| rs13257363 | 8 | 142252580 | G | A | 0.591 | 50 | 0.018 | 0.003 | 0.017 | 0.0068 |
| rs13271368 | 8 | 126506140 | C | T | 0.761 | 47 | 0.02 | 0.003 | 0.0082 | 0.008 |
| rs134594 | 22 | 29468456 | C | T | 0.351 | 42 | 0.017 | 0.003 | 0.0115 | 0.007 |
| rs139429176 | 12 | 121632160 | C | T | 0.988 | 38 | 0.071 | 0.012 | -0.0137 | 0.0396 |
| rs1411424 | 9 | 113892963 | A | G | 0.523 | 64 | 0.02 | 0.003 | -0.0007 | 0.0066 |
| rs1415701 | 6 | 130345835 | G | A | 0.736 | 40 | 0.018 | 0.003 | 0.0104 | 0.0075 |
| rs147110934 | 19 | 55993436 | G | T | 0.975 | 42 | 0.052 | 0.009 | 0.0975 | 0.0256 |
| rs1482852 | 3 | 156798294 | A | G | 0.599 | 386 | 0.05 | 0.003 | 0.0135 | 0.0069 |
| rs1547669 | 6 | 33775641 | G | A | 0.497 | 52 | 0.018 | 0.003 | -0.0162 | 0.0066 |
| rs17034876 | 2 | 46484310 | T | C | 0.7 | 238 | 0.042 | 0.003 | 0.0044 | 0.0075 |
| rs1818782 | 5 | 39424628 | C | A | 0.637 | 38 | 0.016 | 0.003 | -0.0151 | 0.0071 |
| rs186606513 | 2 | 97482001 | G | A | 0.978 | 51 | 0.061 | 0.01 | 0.0323 | 0.0272 |
| rs1937436 | 1 | 66441329 | G | A | 0.71 | 34 | 0.016 | 0.003 | 0.0021 | 0.0073 |
| rs1964859 | 20 | 607805 | T | C | 0.3 | 35 | 0.016 | 0.003 | -0.0049 | 0.0076 |
| rs2045457 | 16 | 20046115 | G | A | 0.311 | 35 | 0.016 | 0.003 | -0.0054 | 0.0071 |
| rs2189234 | 4 | 106075498 | G | T | 0.618 | 34 | 0.015 | 0.003 | 0.0122 | 0.0068 |
| rs220193 | 21 | 43581308 | A | G | 0.225 | 49 | 0.021 | 0.003 | -0.0074 | 0.0087 |
| rs222857 | 17 | 7164563 | T | C | 0.575 | 106 | 0.026 | 0.003 | -0.0079 | 0.0067 |
| rs2229742 | 21 | 16339172 | G | C | 0.881 | 49 | 0.027 | 0.004 | 0.0239 | 0.0113 |
| rs2237467 | 7 | 50733316 | A | G | 0.221 | 36 | 0.018 | 0.003 | 0.0194 | 0.008 |
| rs2238464 | 16 | 2332577 | T | C | 0.321 | 32 | 0.015 | 0.003 | 0.0162 | 0.0071 |
| rs2242116 | 3 | 46941116 | A | G | 0.391 | 34 | 0.015 | 0.003 | 0.0152 | 0.0069 |
| rs2262207 | 13 | 114136110 | A | G | 0.748 | 35 | 0.017 | 0.003 | -0.0075 | 0.0077 |
| rs2280235 | 2 | 191843830 | G | A | 0.259 | 40 | 0.018 | 0.003 | -0.0013 | 0.0077 |
| rs2282978 | 7 | 92264410 | C | T | 0.326 | 46 | 0.018 | 0.003 | -0.0339 | 0.0071 |
| rs2292626 | 10 | 124186714 | T | C | 0.478 | 64 | 0.02 | 0.003 | 0.0064 | 0.0061 |
| rs2306547 | 12 | 26877885 | C | T | 0.534 | 58 | 0.019 | 0.003 | -0.0114 | 0.0067 |
| rs2306700 | 3 | 142123841 | T | C | 0.136 | 40 | 0.023 | 0.004 | 0.0256 | 0.0098 |
| rs2395668 | 6 | 37105893 | A | G | 0.84 | 35 | 0.02 | 0.004 | 0.0108 | 0.0093 |
| rs2551347 | 2 | 23912401 | T | C | 0.749 | 70 | 0.024 | 0.003 | 0.0154 | 0.0077 |
| rs2647873 | 12 | 103081192 | A | G | 0.52 | 52 | 0.018 | 0.003 | 0.0081 | 0.0067 |
| rs2663842 | 18 | 55449516 | A | G | 0.678 | 36 | 0.016 | 0.003 | -0.0087 | 0.0072 |
| rs2747503 | 6 | 15066121 | C | T | 0.663 | 37 | 0.016 | 0.003 | -0.003 | 0.0087 |
| rs2779165 | 19 | 4915447 | G | C | 0.184 | 47 | 0.022 | 0.003 | 0.0142 | 0.009 |
| rs2807319 | 1 | 22554176 | G | A | 0.883 | 35 | 0.023 | 0.004 | 0.0142 | 0.0104 |
| rs28365970 | 5 | 67585723 | C | A | 0.741 | 49 | 0.02 | 0.003 | 0.0104 | 0.0076 |
| rs28457693 | 9 | 98217348 | G | A | 0.109 | 121 | 0.044 | 0.004 | 0.023 | 0.0102 |
| rs28505901 | 9 | 139246588 | A | G | 0.249 | 69 | 0.024 | 0.003 | 0.046 | 0.0194 |
| rs2889874 | 20 | 33715777 | G | T | 0.452 | 41 | 0.016 | 0.003 | 0.0146 | 0.0069 |
| rs2934844 | 6 | 166142456 | T | A | 0.672 | 62 | 0.021 | 0.003 | 0.0159 | 0.0075 |
| rs2946179 | 5 | 157886627 | C | T | 0.734 | 50 | 0.02 | 0.003 | -0.0163 | 0.0075 |
| rs339969 | 15 | 60883281 | A | C | 0.619 | 44 | 0.017 | 0.003 | 0.0099 | 0.0069 |
| rs34036147 | 8 | 38366249 | T | C | 0.688 | 45 | 0.018 | 0.003 | 0.0027 | 0.0074 |
| rs351930 | 5 | 52003397 | T | A | 0.801 | 37 | 0.019 | 0.003 | 0.0051 | 0.0082 |
| rs35261542 | 6 | 20675792 | C | A | 0.733 | 211 | 0.041 | 0.003 | -0.0011 | 0.0075 |
| rs3806315 | 1 | 214724668 | A | G | 0.591 | 50 | 0.018 | 0.003 | -0.0067 | 0.0069 |
| rs3933326 | 9 | 123633948 | G | A | 0.676 | 62 | 0.021 | 0.003 | 0.002 | 0.0072 |
| rs3965156 | 3 | 66484956 | A | C | 0.476 | 36 | 0.015 | 0.003 | 0.0235 | 0.0068 |
| rs40434 | 16 | 55699525 | G | A | 0.391 | 44 | 0.017 | 0.003 | -0.0066 | 0.0068 |
| rs41311445 | 22 | 42070374 | A | C | 0.903 | 61 | 0.033 | 0.004 | 0.0393 | 0.0117 |
| rs41355649 | 19 | 33790556 | G | A | 0.934 | 46 | 0.034 | 0.005 | 0.0131 | 0.0154 |
| rs4144829 | 4 | 17903654 | C | T | 0.267 | 163 | 0.036 | 0.003 | 0.0092 | 0.0075 |
| rs4350272 | 10 | 25056118 | A | G | 0.269 | 37 | 0.017 | 0.003 | 0.0115 | 0.0075 |
| rs4444073 | 11 | 10331664 | A | C | 0.52 | 64 | 0.02 | 0.003 | -0.014 | 0.0067 |
| rs4719648 | 7 | 2756832 | C | T | 0.577 | 57 | 0.019 | 0.003 | 0.0192 | 0.0068 |
| rs4794720 | 17 | 55392223 | G | A | 0.362 | 33 | 0.015 | 0.003 | -0.004 | 0.0069 |
| rs4809731 | 20 | 47495767 | G | C | 0.131 | 35 | 0.022 | 0.004 | -0.011 | 0.01 |
| rs4867699 | 5 | 172748540 | T | G | 0.507 | 36 | 0.015 | 0.003 | -0.001 | 0.0068 |
| rs5030317 | 11 | 32410337 | C | G | 0.733 | 36 | 0.017 | 0.003 | -0.0103 | 0.0075 |
| rs5030938 | 10 | 70975916 | T | C | 0.686 | 80 | 0.024 | 0.003 | -0.0036 | 0.0071 |
| rs516246 | 19 | 49206172 | C | T | 0.506 | 52 | 0.018 | 0.003 | -0.0026 | 0.0068 |
| rs55836809 | 13 | 28502874 | A | G | 0.78 | 36 | 0.018 | 0.003 | 0.0045 | 0.0082 |
| rs55958435 | 15 | 96852638 | A | G | 0.748 | 76 | 0.025 | 0.003 | -0.0034 | 0.0077 |
| rs57414412 | 11 | 111769431 | G | A | 0.719 | 33 | 0.016 | 0.003 | 0.0135 | 0.0074 |
| rs5742915 | 15 | 74336633 | C | T | 0.456 | 36 | 0.015 | 0.003 | 0.017 | 0.0068 |
| rs59084784 | 7 | 22739562 | A | C | 0.323 | 41 | 0.017 | 0.003 | 0.0048 | 0.0072 |
| rs61830764 | 1 | 212289976 | A | G | 0.377 | 44 | 0.017 | 0.003 | -0.0119 | 0.0072 |
| rs62023486 | 15 | 53070589 | A | G | 0.897 | 31 | 0.023 | 0.004 | -0.0085 | 0.011 |
| rs62496903 | 8 | 6446938 | T | C | 0.083 | 53 | 0.033 | 0.005 | -0.0036 | 0.0131 |
| rs62562580 | 9 | 94252219 | G | C | 0.212 | 31 | 0.017 | 0.003 | 0.0216 | 0.0081 |
| rs6575803 | 14 | 101257755 | C | T | 0.895 | 62 | 0.032 | 0.004 | -0.0023 | 0.0115 |
| rs6582623 | 12 | 46613394 | C | T | 0.869 | 42 | 0.024 | 0.004 | 0.0126 | 0.01 |
| rs667515 | 11 | 69449076 | G | C | 0.618 | 49 | 0.018 | 0.003 | 0.0148 | 0.0071 |
| rs6845999 | 4 | 145565826 | T | C | 0.431 | 107 | 0.026 | 0.003 | 0.01 | 0.0067 |
| rs6871635 | 5 | 133830395 | G | A | 0.566 | 40 | 0.016 | 0.003 | -0.0093 | 0.0068 |
| rs6925689 | 6 | 126865884 | T | C | 0.494 | 36 | 0.015 | 0.003 | 0.0047 | 0.0067 |
| rs6930558 | 6 | 141878920 | T | G | 0.747 | 59 | 0.022 | 0.003 | 0.0071 | 0.0077 |
| rs6958858 | 7 | 148966949 | C | T | 0.487 | 31 | 0.014 | 0.003 | -0.0029 | 0.0067 |
| rs7075355 | 10 | 82208878 | A | G | 0.533 | 36 | 0.015 | 0.003 | -0.0132 | 0.0067 |
| rs7076938 | 10 | 115789375 | T | C | 0.735 | 128 | 0.032 | 0.003 | 0.0103 | 0.0075 |
| rs708122 | 1 | 228216997 | C | A | 0.681 | 40 | 0.017 | 0.003 | -0.0061 | 0.0072 |
| rs7102454 | 11 | 65594820 | C | T | 0.353 | 33 | 0.015 | 0.003 | -0.0037 | 0.007 |
| rs7183988 | 15 | 91428589 | G | T | 0.529 | 52 | 0.018 | 0.003 | -0.0069 | 0.0068 |
| rs7223535 | 17 | 29211667 | G | A | 0.732 | 56 | 0.021 | 0.003 | 0.0262 | 0.0076 |
| rs72656010 | 8 | 57122215 | T | C | 0.868 | 58 | 0.028 | 0.004 | 0.0003 | 0.0101 |
| rs73143584 | 20 | 62445702 | A | G | 0.11 | 53 | 0.029 | 0.004 | 0.0022 | 0.0113 |
| rs732563 | 8 | 23345526 | C | T | 0.504 | 46 | 0.017 | 0.003 | 0.0014 | 0.0066 |
| rs73354194 | 17 | 79905947 | C | T | 0.025 | 58 | 0.061 | 0.009 | 0.0352 | 0.024 |
| rs7525870 | 1 | 78269207 | G | A | 0.74 | 32 | 0.016 | 0.003 | 0.0017 | 0.0075 |
| rs754868 | 2 | 43185532 | G | A | 0.419 | 40 | 0.016 | 0.003 | -0.0109 | 0.0067 |
| rs75518158 | 21 | 38393567 | C | A | 0.033 | 33 | 0.04 | 0.007 | -0.0044 | 0.0187 |
| rs7563664 | 2 | 158344455 | T | G | 0.107 | 35 | 0.024 | 0.004 | 0.0021 | 0.011 |
| rs75844534 | 15 | 38667117 | A | C | 0.124 | 47 | 0.026 | 0.004 | 0.0097 | 0.0107 |
| rs76094073 | 6 | 109288036 | G | C | 0.121 | 50 | 0.027 | 0.004 | 0.0114 | 0.0101 |
| rs76895963 | 12 | 4384844 | G | T | 0.021 | 76 | 0.076 | 0.01 | 0.1564 | 0.0296 |
| rs7709066 | 5 | 77831071 | C | T | 0.455 | 31 | 0.014 | 0.003 | -0.0117 | 0.0067 |
| rs7744700 | 6 | 53349401 | T | A | 0.711 | 53 | 0.02 | 0.003 | 0.0091 | 0.0076 |
| rs77553582 | 7 | 35299657 | T | C | 0.613 | 128 | 0.029 | 0.005 | -0.0446 | 0.0092 |
| rs7819593 | 8 | 106115172 | C | T | 0.243 | 57 | 0.022 | 0.003 | 0.0067 | 0.0077 |
| rs80019595 | 12 | 121417306 | T | C | 0.027 | 34 | 0.045 | 0.008 | -0.0172 | 0.0236 |
| rs80278614 | 1 | 119412317 | A | G | 0.054 | 53 | 0.04 | 0.006 | 0.0138 | 0.0151 |
| rs8038207 | 15 | 86316570 | G | T | 0.569 | 35 | 0.015 | 0.003 | 0.0062 | 0.0067 |
| rs8106042 | 19 | 7161849 | G | C | 0.281 | 52 | 0.02 | 0.003 | -0.0025 | 0.0078 |
| rs8756 | 12 | 66359752 | C | A | 0.487 | 270 | 0.041 | 0.003 | 0.0154 | 0.0066 |
| rs905938 | 1 | 154991389 | C | T | 0.262 | 84 | 0.026 | 0.003 | 0.0432 | 0.0076 |
| rs9318511 | 13 | 78601413 | C | A | 0.873 | 52 | 0.027 | 0.004 | -0.0019 | 0.0104 |
| rs9348981 | 6 | 35687249 | T | G | 0.71 | 58 | 0.021 | 0.003 | 0.0072 | 0.0074 |
| rs9366778 | 6 | 31269173 | G | A | 0.627 | 49 | 0.018 | 0.003 | 0.0118 | 0.0074 |
| rs9549046 | 13 | 40647206 | A | G | 0.118 | 56 | 0.029 | 0.004 | -0.0025 | 0.0102 |
| rs962554 | 6 | 142734204 | T | C | 0.715 | 38 | 0.017 | 0.003 | 0.0165 | 0.0073 |
| rs9783782 | 16 | 88317285 | G | T | 0.312 | 35 | 0.016 | 0.003 | 0.0106 | 0.0078 |

SNP, single-nucleotide polymorphism; Chr, chromosome; Pos, position; EA, effect allele; OA, other allele; EAF, frequency of effect allele; SE, standard error.

**Table S4.** Power calculation for the primary analysis.

| Factors | Settings |
| --- | --- |
| Sample size | 1030836 |
| Type-I error rate | 0.05 |
| Proportion of AF cases | 0.06 |
| Odds ratio of AF per standard deviation of BW | 1.27 |
| Proportion of variance explained for the association between the 144 SNPs and BW | 0.03 |
| Power | 1.00 |

AF, atrial fibrillation; BW, birth weight; SNP, single nucleotide polymorphism.

**Figure legends.**

**Figure S1.** IVW analysis of the causal association between birth weight and atrial fibrillation. The black dots indicate the estimates of the causal effect using the single SNP, and the black bar indicate the relevant 95% confidence interval. The red dot and bar indicate the overall estimate and the 95% confidence interval using the IVW method. IVW, inverse variance-weighted; SNP, single nucleotide polymorphism.

**Figure S2.** Scatter plot of the association of each SNP with birth weight and its effect on atrial fibrillation. The dots and bars indicate the estimates and 95% confidence interval using each SNP. The line indicates the association between the genetic predicted birth weight and the risk of atrial fibrillation using various statistical models. SNP, single nucleotide polymorphism.

**Figure S3.** Leave-one-out analysis of the causal association between birth weight and atrial fibrillation. The black dots and bars indicate the estimates and 95% confidence interval when the specific SNP is removed. The red dot and line indicate the overall estimate and 95% confidence interval using the IVW method. SNP, single nucleotide polymorphism; IVW, inverse variance-weighted.

**Figure S4.** The association between birth weight and body mass index investigated with different statistical methods. OR, odds ratio; CI, confidence interval; MR, Mendelian randomization; MR-PRESSO, Mendelian Randomization Pleiotropy Residual Sum and Outlier.

**Figure S1.**

**Figure S2.**

**Figure S3.**

**Figure S4.**

**References**

1. Warrington NM, Beaumont RN, Horikoshi M, et al. Maternal and fetal genetic effects on birth weight and their relevance to cardio-metabolic risk factors. *Nat Genet* 2019; 51: 804–814.

2. Nielsen JB, Thorolfsdottir RB, Fritsche LG, et al. Biobank-driven genomic discovery yields new insight into atrial fibrillation biology. *Nature Genetics*. Epub ahead of print 2018. DOI: 10.1038/s41588-018-0171-3.
